# Can the concept of Health Promoting Schools help to improve students' health knowledge and practices to combat the challenge of communicable diseases: Case study in Hong Kong?

**DOI:** 10.1186/1471-2458-8-42

**Published:** 2008-01-30

**Authors:** Albert Lee, Martin CS Wong, Vera MW Keung, Hilda SK Yuen, Frances Cheng, Jennifer SY Mok

**Affiliations:** 1 Centre for Health Education and Health Promotion, The Chinese University of Hong Kong, Hong Kong, China; 2 Department of Community and Family Medicine, The Chinese University of Hong Kong, Hong Kong, China

## Abstract

**Background:**

The growing epidemics of emerging infectious diseases has raised the importance of a setting approach and include the Health Promoting School (HPS) framework to promote better health and hygiene. Built on the concept of 'the' HPS framework, the Hong Kong Healthy Schools Award scheme includes "Personal Health Skills" as one of its key aspects to improve student hygiene knowledge and practices. This study examines the differences in student perceptions, knowledge and health behaviours between those schools that have adopted the HPS framework and those that have not adopted.

**Methods:**

A cross-sectional study using multi-stage random sampling was conducted among schools with awards (HSA) and those schools not involved in the award scheme nor adopting the concept of HPS (non-HPS). For HSA group, 5 primary schools and 7 secondary schools entered the study with 510 students and 789 students sampled respectively. For the 'Non-HPS' group, 8 primary schools and 7 secondary schools entered the study with 676 students and 725 students sampled respectively. A self-administered questionnaire was used as the measuring instrument.

**Results:**

Students in the HSA category were found to be better with statistical significance in personal hygiene practice, knowledge on health and hygiene, as well as access to health information. HSA schools were reported to have better school health policy, higher degrees of community participation, and better hygienic environment.

**Conclusion:**

Students in schools that had adopted the HPS framework had a more positive health behaviour profile than those in non-HPS schools. Although a causal relationship is yet to be established, the HPS appears to be a viable approach for addressing communicable diseases.

## Background

Health behaviours are strongly determined by the different social, economic and environmental circumstances of individuals and populations. Improvement of health literacy can help individuals to tackle the determinants of health better as it builds up the personal, cognitive and social skills which determine the ability of individuals to gain access to, understand and use of information to promote and maintain good health [1]. Schools are essential in helping students to achieve health literacy [2]. The concept of the **health promoting school **(HPS) has been advocated as an effective approach to promote health in schools [3,4]. It embodies a holistic, whole school approach in which a broad health education curriculum is supported by the environment and ethos of the school and shifts health into a more dynamic and political domain to address the determinants of heath [2,5]. Healthy Schools Award schemes are very popularamong the European countries as structured frameworks acting as systems for monitoring and recognition of achievements [6]. Moon has shown positive award-related changes in terms of children's health behaviours, and that the awarded schools have more health promoting culture and organization [7].

The outbreak of Severe Acute Respiratory Syndrome (SARS) has highlighted the importance of maintaining a healthy and hygienic environment as one of the effective public health measures to combat infectious diseases, but the major challenge is that some of the most important public health measures are to be taken outside the health sector [8]. Schools have become an important setting to build up the skills and capacity for students, parents and the wider community to combat the challenges of outbreak of communicable diseases [9]. Rosen et al has demonstrated that a hygiene program can produce a sustained increase in hand washing rates among toilet-trained preschool children [10]. Hand washing has been shown to be an effective means to prevent spread of infection [11]. These findings illuminate the potential of schools as promising venues for promoting hygiene and health in prevention of infectious diseases.

The Centre for Health Education and Health Promotion, The Chinese University of Hong Kong (CHEP) launched the first territory-wide "Healthy Schools Award Scheme" (HSA) in 2001 [12]. It was modeled on the World Health Organization (WHO) Regional Office of the Western Pacific Health Promoting School (HPS) framework covering six key areas (health policy, physical and social environments, community relationships, personal health skills and health services) designed to assist schools in addressing particular health issues strategically [13-16]. The scheme has developed a set of indicators and guidelines to meet the local needs. Each key area has a number of components with targets for the schools to achieve [13-15]. The participating schools attended training programme on development planning at the start of the project helping them to appreciate the breadth of the scheme and consolidate their thoughts for appropriate action. The HSA project team met with the co-ordinating group of the school to identify and prioritize four to five areas of potential action. The project team visited the school at least once per term to offer advice and support and obtained detailed information on each of the activities for evaluation. The amount of support given varied from area to area according to the needs of the schools and the summary of health promoting activities have been described in the special issues [17-19].

The process of selection of indicators for the scheme and also the accreditation process have incorporated advice and validation by a number of local and international experts in the field [15,20]. A practical manual with detailed guidelines was developed for participating schools [13,14]. "Personal health skills" is one key component, where basic knowledge and skills in hygienic measures and prevention of communicable diseases are incorporated in a variety of school activities like classroom teaching, interactive learning initiatives, community-wide health promoting campaigns, and teaching staffs are equipped with relevant training and resources [9,13-15,20].

While process evaluations showed that health promoting activities had been successfully implemented in schools, the evaluation of the effectiveness of HPS reported a wide range of outcomes [21-23]. This survey explores the differences in student perceptions, knowledge and health behaviours between schools that have adopted the HPS framework and those non-HPS. The Education Authority in Hong Kong recommended using the concept of HPS to improve school health and hygiene [24,25] with the hypothesis that children in HPS schools more likely to perceive that their school is a healthier place. The result would have significant impact on the future planning, implementation and substantiation of HPS in different parts of the World as the problem of emerging infectious diseases becomes a global issue.

## Method

### Subjects and sampling

A cross-sectional study was conducted in two categories of schools, namely HSA and non-HPS by multi-stage random sampling.

At the time of the study there were 24 primary and 17 secondary schools that had obtained awards for HPS. For comparison between the two categories of schools, assuming the odds ratio of 2 with the non-HPS school having prevalence of 30% of parents participated in school cleaning activities, a sample size of 240 were needed for each group (N = {1.28 × [P_1 _× (1-P_1_) + P_2 _× (1-P_2_)]^-2 ^+ 1.96 × [2 P_0_(1-P_0_)]^-2^}^2^/(P_2 _- P_1_)^2 ^P1 = proportion of control, P_2 _= P_1 _× OR/1 + P_1_(OR-1); P_0 _= P_1 _+ P_2_/2) [26]. Random sampling method was used to target at least 500 students from primary and secondary schools in HSA category (double the required numbers) and 750 non-HPS category (triple the required numbers) from primary and secondary schools. Schools were randomly selected from each category respectively and letters of invitation were sent out in lots of tens to the selected schools until the targeted numbers of students had been reached.

The first stage consisted of random selection by category and the selected schools were invited by letters. In the second stage, we stratified the participating schools by grades, with samples from Primary 4 (P4) to Primary 6 (P6) (aged around 10–12) and Secondary 1 (S1) to Secondary 3 (S3) (aged around 13–15). The third stage was a systematic random sampling, where one class of students was randomly selected from each grade to participate in the survey.

### Measures

The questionnaire testing health-related knowledge and hygienic practice was devised by the research team in CHEP based on evaluation tools used by Hong Kong HSA [12,15,20] and the content was further validated by two medical doctors with postgraduate degrees in Public Health. Health-related knowledge was reported as a score for each student, and other outcome measures adopt an ordinal scale (e.g. "very clean", "clean", "not quite", "not clean"), which was then grouped into binary variables (e.g. "very clean" and "clean" as a group vs. those not) for statistical analysis. The complete set of questionnaire had been pilot tested in a primary school for face-validity and reliability with good results. The items are well understood. The 2-week test-retest reliability has shown all the items with kappa statistic 0.7 or higher.

The following aspects were examined in the survey:

• Demographic data

• Personal hygienic practices at school, home and public areas

• Students' perception of school health policy

• Community participation by schools

• School physical environment

• Students' knowledge on health and hygiene

• Personal health habits

### Data collection

Data collection was done between May and June of 2005. For the HSA group, 510 students and 789 students were sampled from 5 primary schools and 7 secondary schools respectively. For the 'Non-HPS' group, 676 students and 725 students were sampled from 8 primary schools and 7 secondary schools respectively. The response rate from students of the participating schools was nearly 100% for all the schools (except those absent from schools). Letters were sent out to students and parents to obtain consent for participation on voluntarily basis. The questionnaires were collected without identifiers to ensure confidentiality. It took about 30 minutes to complete the questionnaire. No refusal was noted from parents and students. The study has been approved by Survey and Behaviour Research Ethics Committee of the Chinese University of Hong Kong

### Data analysis

Proportions of students with particular behaviours and perception of school environment were tabulated. Chi square statistics was used to analyse the difference in proportions between HSA and non-HPS. For knowledge, scores were calculated for each category and t test statistics was used to analyse the difference.

## Results

### Demographic Profiles

The sample consisted of approximately equal gender distribution (see Table 1). The majority of students (around 79%) were born in Hong Kong, with a minority born in Mainland (21%) or other countries. The mean age of the participants from secondary schools was 14.46 (S.D. ± 1.33) and 14.51 (S.D. ± 1.25) year-old in HSA and non-HPS schools respectively. The mean age of the participants from primary schools was 11.15 (S.D. ± 0.95) and 11.54 (S.D. ± 1.56) year-old in HSA and non-HPS schools respectively.

**Table 1:** Grade and Gender of the respondents by school category

***Primary schools***	**HSA (n = 510)**	**Non-HPS (n = 676)**
P.4	165(32.4%)	228 (33.7%)
P.5	168(32.9%)	217 (32.1%)
P.6	177(34.7%)	231 (34.2%)
Boy	242(47.6%)	359 (53.3%)
Girl	266(52.4%)	315 (46.7%)

***Secondary schools***	**HSA (n = 789)**	**Non-HPS (n = 725)**

S.1	260(33.0%)	217 (29.9%)
S.2	273(34.6%)	260 (35.9%)
S.3	256(32.4%)	248 (34.2%)
Boy	367(46.9%)	308 (42.6%)
Girl	415(53.1%)	415 (57.4%)

(Note: A minority of the students missed filling in their demographic data)

**HSA**: awarded schools in the HSA Scheme

**Non-HPS **schools are those not in the HSA scheme and whose teachers did not attend any training in HPS or diplomas

### Personal hygienic practices and skills at school, home and public areas

#### Handwashing practices

In terms of hand washing habits after toileting, only small difference was detected as this is something that has been enmeshed into the children's everyday life especially since the SARS epidemic.

There was a significant difference between HSA schools and non-HPS schools in teachers reminding students to wash their hand before meals (Table 2). Also higher proportion of secondary school students washed hands with their own initiatives before meals although the reverse was observed amongst primary students (Table 2)

**Table 2:** Personal hygienic practices and skills at school, home and public areas

	**HSA**	**Non-HPS**	**P value**
**1. Handwashing Before meal always**			
Primary	60.4% (307/508)	66.1% (446/675)	**0.05**
Secondary	46.6% (366/785)	40.1% (291/725)	**0.01**
**2. Handwashing after toileting always**			
Primary	94.9% (482/508)	92.6% (624/674)	0.11
Secondary	93.6% (737/787)	93.2% (676/725)	0.75
**3. Teachers reminded students to wash hands before meal**			
Primary	65.2% (332/509)	75.9% (491/647)	**<0.001**
Secondary	31.9% (251/786)	8.3% (60/722)	**<0.001**
**4. Brush teeth after meal always**			
Primary	28.2% (143/507)	19.7% (133/675)	**0.001**
Secondary	10.7% (84/783)	12.4% (90/724)	0.30
**5. Bath everyday**			
Primary	99.6% (506/508)	98.1% (663/676)	**0.02**
Secondary	98.9% (777/786)	98.3% (710/722)	0.39
**6. correct coping when fever**			
Primary	80.9% (412/509)	75.2%(507/674)	**0.02**
Secondary	64.0% (497/777)	58.0%(418/721)	**0.02**

#### Tooth Brushing and Bathing

Table 2 shows that in primary schools, higher proportion of students from HSA schools (28.2%) *always *brushed their teeth after meal than the non-HPS category (19.7%) with statistical significance (p = 0.001) (Table 2).

The majority of students had a bath everyday but HSA primary school students reported a significantly higher proportion than non-HPS schools (99.6% vs. 98.1%, p = 0.02; table 2).

#### Correct self care with fever

Higher proportion of students from HSA schools in both primary and secondary schools (80.9% vs 75.2% and 64.0% vs 58.0% respectively) coped correctly when they had fever with statistical significance (p = 0.02; Table 2).

### School health policy

There was no significant difference amongst primary school children encouraged to wear face mask or staying away from school if they had symptoms of Upper Respiratory Tract Infection (URTI). However difference was observed in mask wearing between the HSA and non-HPS secondary schools (86.8% vs 80.4%) and in staying away from schools if students had URTI symptoms (93.6% vs 90.1%) with statistical significance (p = 0.001 and p = 0.01 respectively, Table 3).

**Table 3:** Students' perception of school health policy

	**HSA**	**Non-HPS**	**p value**
**1. Schools encourage face mask wearing when URTI**			
Primary	88.4% (450/509)	88.0% (595/676)	0.84
Secondary	86.8%(681/785)	80.4% (582/724)	**0.001**
**2. Schools tell student to stay away from school when URTI**			
Primary	96.7% (491/508)	95.5% (644/674)	0.34
Secondary	93.6%(737/787)	90.1% (652/724)	**0.01**
**3. Schools mention hygiene charter website**			
Primary	30.6% (156/509)	30.8% (207/671)	0.94
Secondary	28.1% (221/786)	15.3% (111/724)	**<0.001**

The Hygiene Charter, launched in 2003 as a community initiative supported by various business and industry sectors to pledge their long-term commitment to fight against the challenges posed by SARS and create a healthy and hygienic environment for Hong Kong [23]. Its website is of high reference value for schools. In secondary schools, higher proportion of students from the HSA group reported that teachers had mentioned the Hygiene Charter website (28% vs 15.3%; p < 0.001) (Table 3).

### Community participation by schools

In secondary schools, higher proportion of students from the HSA group (23.6% vs 19.2%) felt that their schools joined community cleaning with statistical significance (p = 0.04) (Table 4). The organization of activities to promote health and hygiene was also reported by higher proportion of students from the HSA than the non-HPS group in both primary (60.9% vs. 56.7%) and secondary schools (53.7% vs. 37.0%, p < 0.001) (Table 4). Significantly higher proportion of parents from the HSA group joined school cleaning activities in both primary and secondary schools (42.7% vs. 31.9%; p < 0.001, and 20.3% vs. 8.6%; p < 0.001 respectively) (Table 4).

**Table 4:** Community Participation and Physical Environment

	**HSA**	**Non-HPS**	**p value**
**1. Schools Join community cleaning**			
Primary	43.9% (219/509)	43.1% (291/675)	0.97
Secondary	23.6% (186/787)	19.2% (139/725)	**0.04**
**2. school organize health promoting activities**			
Primary	60.9% (310/509)	56.7% (382/674)	0.14
Secondary	53.7% (422/786)	37.0% (268/724)	**<0.001**
**3. Parents join school cleaning activities**			
Primary	42.7% (217/508)	31.9% (215/675)	**<0.001**
Secondary	20.3% (160/787)	8.6% (62/723)	<0.001
**4. Playgrounds perceived as very clean or clean**			
Primary	72.2% (366/507)	61.6% (415/674)	**<0.001**
Secondary	56.5% (444/786)	53.5% (386/721)	0.25
**5. Classrooms perceived as very clean or clean**			
Primary	73.6% (374/508)	68.5% (462/674)	0.058
Secondary	51.3% (404/787)	52.5% (380/724)	0.65
**6. Toilets perceived as very clean or clean**			
Primary	34.7% (176/507)	38.5% (259/673)	0.18
Secondary	29.7% (233/784)	27.6% (199/721)	0.36
**7. Staircases perceived as very clean or clean**			
Primary	77.2% (392/508)	76.2% (513/673)	0.71
Secondary	68.7% (540/786)	63.3% (457/722)	**0.03**
**8. Tuckshop perceived as very clean or clean**			
Primary	84.1% (428/509)	81.4% (407/500)	0.26
Secondary	60.2% (471/783)	53.3% (313/587)	**0.01**
**9. Prefect for cleaning**			
Primary	75.0% (382/509)	64.4% (435/675)	**<0.001**
Secondary	52.5% (412/785)	34.2% (247/723)	**<0.001**
**10. Prefects for environment Protection**			
Primary	63.1% (321/509)	59.7% (403/675)	0.24
Secondary	49.4% (388/786)	23.7% (171/723)	**<0.001**

### School physical environment

Primary school students in the HSA group had higher proportion choosing ''very clean'' or ''clean'' for playground hygiene (72.2%) as compared to 61.6% in the non-HPS group (p < 0.001; Table 4). For classroom 73.6% of students from HSA schools chose ''very clean'' or ''clean'' compared with 68.5% in non-HPS schools (p = 0.058) (Table 4). Secondary school students in HSA group had higher proportion choosing ''very clean'' or ''clean'' for stairs (68.7% vs 63.3% p = 0.03) and tuck shop (60.2% vs 53.3% p = 0.01) (Table 4).

More HSA primary (75.0% vs. 64.4%; p < 0.001) and secondary (52.5% vs. 34.2%, p < 0.001) students perceived their schools having assigned prefects to monitor or help with cleaning with statistical significance. More HSA primary school students reported having prefects for environmental protection (63.1% vs. 59.7%), and similarly in secondary schools (49.4% vs. 23.7%) with statistical significance (p < 0.001) (Table 4).

### Literacy on health and hygiene

In most areas of health-related knowledge, it was found that the majority of students in both groups have ever acquired related knowledge. There was no significant difference amongst the secondary school students between the HSA and non-HPS groups in the overall scores for both categories (12.40 vs. 12.41; p = 0.95). However in primary schools, the difference in overall scores between the two groups was found to be statistically significant (12.13 vs 11.58; p < 0.001) (Table 5).

**Table 5:** Literacy on health and hygiene

	**HSA**	**Non-HPS**	**p value**
**1. Health knowledge scores (mean ± SD)**			
Primary	12.13+2.45	11.58+2.82	**<0.001**
Secondary	12.40+2.44	12.41+2.69	0.95
**2. get health info from teachers (very much or quite a lot)**			
Primary	86.7% (441/509)	84.2%(568/674)	0.25
Secondary	69.4% (546/786)	68.7%(498/725)	0.74
**3. Get health info from classmates (very much or quite a lot)**			
Primary	39.4% (200/508)	38.8%(260/671)	0.83
Secondary	28.4% (223/786)	26.6%(193/725)	0.45
**4. Get health Information from ambassador (very much or quite a lot)**			
Primary	32.8% (165/503)	17.7%(119/673)	**<0.001**
Secondary	19.0% (149/785)	10.5% (76/720)	**<0.001**
**5. Get health info from media (very much or quite a lot)**			
Primary	46.5% (236/507)	43.2%(291/673)	**0.002**
Secondary	48.5% (380/783)	49.8%(360/724)	0.64
**6. Get health info from pamphlets (very much or quite a lot)**			
Primary	52.3% (265/507)	46.2%(311/674)	**0.04**
Secondary	36.5% (287/786)	27.3%(197/722)	**<0.001**
**7. Get health info from website (very much or quite a lot)**			
Primary	42.3% (215/508)	44.9%(303/674)	0.37
Secondary	20.3% (160/786)	19.7%(143/724)	0.77

Higher proportion of primary school students in HSA group had chosen "very much" or "quite a lot" in getting health information from student health ambassadors (32.8% vs 17.7%; p < 0.001), media (46.5% vs. 43.2%; p = 0.002), and pamphlets (52.3% vs 46.2% p = 0.04) with statistical significance (Table 5). For secondary schools, it was also found that higher proportion of students in HSA group had chosen "very much" or "quite a lot" in getting health information from student ambassadors (19.0% vs 10.5%; p < 0.001) and pamphlets (36.5% vs 27.3% p < 0.001) with statistical significance (Table 5).

## Discussion

On the whole, students from the HSA group showed positive outcomes in most aspects of health and hygiene particularly the hygiene practice of students and actions taken by schools. It is not unexpected as HPS embodies a holistic, whole school approach to personal and community health promotion [27]. The Hong Kong Healthy Schools Award scheme builds on the concept of HPS which helps to create a supportive environment for healthy development in schools, home and the community [6]. Studies have shown that students from schools which had comprehensively embraced the HPS concept as indicated by attaining the 'Healthy School Award', were better, in terms of health and educational outcomes, than students from schools that did not reach the standard of the award [28,29].

St Leger and Nutbeam [30] stated that HPS contributes to four school related outcomes. They are: (a) lifelong learning; (b) competencies and behaviors; (c) specific cognate knowledge and skills; and (d) self attributes. Therefore HSA schools would confer a great impact on school health and hygiene, in addition to students' health habits, as was demonstrated in the present study.

Positive changes were observed in certain aspects of school health and hygiene particularly in getting relevant health information from various reliable sources amongst secondary students in the HSA group. Previous studies have indicated the potential for a settings approach to school health, like the Hong Kong HSA scheme (HKHSA), to provide a promising strategic framework allowing health literacy outcome to be achieved [2,31]. This group of schools also established actions to promote better health and hygiene as the schools participated in community cleaning activities and the parents also supported the schools in cleaning activities. This study reveals that these HSA schools have also organized more health promotion activities. This moves to a more collaborative community-based approach for students to learn, as opposed to a teacher-dominated school hierarchy [2], is one of the strategies to achieve critical health literacy [1].

School-based implementation of HPS was shown to be associated with significant greater levels of positive personal health habits. Although this study is cross-sectional and no cause and effect can be established, it would still highlight how HKHSA might exert its influence individually as well as the school level. Although some of the significant findings of this study is marginal, HPS has only been implemented over a short duration in Hong Kong. HPS programme has been shown to be effective if the intervention is intensive over a long duration [32]. Recent evidence suggests that the way the school is lead and managed, students' experiences in participating and taking responsibility for shaping policies, practices and procedures, teachers-students relationship and school engagement with its local community including parents actually builds many protective factors for health and minimises risk taking behaviour [32-35]. Many of these gains have occurred without a specific health 'intervention'. It appears that a whole school approach to build and maintain a caring school social environment might be the most effective way in achieving good health outcomes.

However putting the HPS in practice takes considerable amount of time and efforts and effective implementation requires commitment and support of various schools' stakeholders. The success of HPS depends heavily on teachers' training like Health Education diploma programmes, which could introduce the concepts to the teachers so they can start making improvements on some fundamental areas of school health and hygiene [36,37]. With time and more teachers being exposed to the concept, teachers would then gradually develop their schools to become HPS. The primary school students in HSA group have shown to have better knowledge scores. Although the difference in knowledge scores between two groups is not statistically significant in secondary schools, it is well known fact that there are other factors in adolescents leading to behaviour and practice change apart from knowledge and belief.

There are some limitations of this study. Schools with more health conscious students or their families might be more willing to adopt HPS. The study design might not be able to control for that history which would have some threat to internal validity. It is a cross sectional survey so it might not capture the longer-term impact of HPS on health behaviours of students due to the different components of HSA interventions. The differences observed might or might not be due to the effect of intervention and future longitudinal studies are needed to ascertain the causal effect fostered by HSA. Furthermore, some outcomes were reported by students only and may involve recall in the past few months, and the effect of multiple comparisons should be taken into account when interpreting statistical significance. In addition, we assumed that randomized sampling minimized the heterogeneity of different clusters and demographic characteristics among schools, which have not been controlled for. This study did not comprehensively assess all the outcomes of HPS as there are different types of outcomes which require different methods of data collection. [14] A descriptive design was adopted instead of randomised control study design because one cannot randomly select schools from all schools in Hong Kong as intervention group since schools need to comply with the standards of HPS to make it an effective programme.

## Conclusion

The study has demonstrated that schools involved in HPS activities through the HSA scheme in Hong Kong had more favorable hygiene practice of students, school environment and atmosphere in health and hygienic practice. Although all schools in Hong Kong have health elements in the curriculum, the primary objective of HPS is to modify the behaviours of school students rather than information giving, so more positive changes are observed in those schools that participated and met the standards in the HSA scheme. These findings highlight the potential of HPS in equipping schools to handle public health crisis such as SARS [9]. Therefore it is highly recommended that schools would further enhance the practice of HPS to empower their students, staffs and parents to enhance preparedness of possible future epidemics of communicable diseases. [38].

## Competing interests

The author(s) declare that they have no competing interests.

## Authors' contributions

Contributions by different authors are as followings-:

- AL and MW were responsible for conception and design, analysis and interpretation of data, drafting and revising the manuscript and gave the final approval

- FC, HY and VK were responsible for conception and design, analysis and interpretation of data, and gave the final approval

- JM was responsible for conception and design, revising the manuscript and gave the final approval

## Pre-publication history

The pre-publication history for this paper can be accessed here:


